# Symbolic Numerical Distance Effect Does Not Reflect the Difference between Numbers

**DOI:** 10.3389/fpsyg.2017.02013

**Published:** 2017-11-16

**Authors:** Attila Krajcsi, Petia Kojouharova

**Affiliations:** ^1^Department of Cognitive Psychology, Institute of Psychology, ELTE Eötvös Loránd University, Budapest, Hungary; ^2^Doctoral School of Psychology, ELTE Eötvös Loránd University, Budapest, Hungary

**Keywords:** symbolic number processing, numerical distance effect, analog number system, discrete semantic system

## Abstract

In a comparison task, the larger the distance between the two numbers to be compared, the better the performance—a phenomenon termed as the numerical distance effect. According to the dominant explanation, the distance effect is rooted in a noisy representation, and performance is proportional to the size of the overlap between the noisy representations of the two values. According to alternative explanations, the distance effect may be rooted in the association between the numbers and the small-large categories, and performance is better when the numbers show relatively high differences in their strength of association with the small-large properties. In everyday number use, the value of the numbers and the association between the numbers and the small-large categories strongly correlate; thus, the two explanations have the same predictions for the distance effect. To dissociate the two potential sources of the distance effect, in the present study, participants learned new artificial number digits only for the values between 1 and 3, and between 7 and 9, thus, leaving out the numbers between 4 and 6. It was found that the omitted number range (the distance between 3 and 7) was considered in the distance effect as 1, and not as 4, suggesting that the distance effect does not follow the values of the numbers predicted by the dominant explanation, but it follows the small-large property association predicted by the alternative explanations.

## The numerical distance effect and its explanations

In a symbolic number comparison task, performance is better (i.e., error rates are lower and reaction times are shorter) when the numerical distance is relatively large, e.g., comparing 1 vs. 9 is easier than comparing 5 vs. 6 (Moyer and Landauer, 1967). There are several explanations for this phenomenon termed the numerical distance effect.

According to the dominant model, numbers are stored on a continuous (analog) and noisy representation called the Analog Number System (ANS). The numbers are stored as noisy signals, and the closer the two numbers on the ANS, the larger the overlap of the two respective signal distributions is. As comparison performance is better when the overlap is relatively small, the large distance number pairs are easier to process because of the smaller overlap between the signals (Dehaene, 2007). More specifically, the ANS works according to Weber's law; therefore, the comparison performance depends on the ratio of the two numbers to be compared (Moyer and Landauer, 1967). In fact, the distance effect is the consequence of this ratio effect because larger distance also means higher ratio. The ratio effect is also thought to be the cause of the numerical size effect: Comparison performance is better for smaller numbers than for larger numbers because smaller number pairs have larger ratio than larger number pairs with the same distance (Moyer and Landauer, 1967). The ANS is thought to be the essential base of numerical understanding (Dehaene, 1992), and numerical distance effect is believed to be a diagnostic signal of the ANS activation while solving a numerical task.

However, there could be another explanation for the cause of the distance effect. Recently, it has been proposed that symbolic numerical effects, such as the distance and size effects, can be explained by a representation similar to the mental lexicon or conceptual networks, where nodes of the network represent the digits, and connections between them are formed according to their semantic and statistical relations (Krajcsi et al., 2016). In this model, termed the Discrete Semantic System (DSS) model, the numerical distance and size effects are rooted in two different mechanisms, even if the combination of these effects looks similar to the formerly supposed ratio effect. According to the model, the size effect might depend on the frequencies of the numbers: Smaller numbers are more frequent than larger numbers (Dehaene and Mehler, 1992); therefore, smaller numbers are easier to process, producing the numerical size effect. A similar frequency-based explanation of the size effect could be found in the model of Verguts et al. (2005). At the same time, numerical distance effect could be based on the relations of the numbers, for example, similar to the phenomenon in a picture naming task, where priming effect size depended on the semantic distance between the prime and target pictures (Vigliocco et al., 2002). There are several other alternative number processing models with partly overlapping suppositions and predictions as the DSS model (Nuerk et al., 2004; Verguts and Fias, 2004; Verguts et al., 2005; Proctor and Cho, 2006; Leth-Steensen et al., 2011; Pinhas and Tzelgov, 2012; Verguts and Van Opstal, 2014). See the comparison of those models in Krajcsi et al. (2016) and in Krajcsi et al. (in press). Supporting the alternative DSS model, it has been found that the size effect followed the frequency of the digits in an artificial number notation comparison task (Krajcsi et al., 2016). In addition, it has been shown in a correlational study that in symbolic number comparison task, the distance and the size effects were independent (Krajcsi, 2017), reflecting two independent mechanisms generating the two effects. (See a similar prediction for independent distance and size effects in Verguts et al., 2005; Verguts and Van Opstal, 2014).

Because of the DSS model and the empirical findings demonstrating that the size effect is a frequency effect and that the distance and size effects are independent, it is essential to reconsider how the distance effect is generated. According to the DSS model, different explanations consistent with the supposed network architecture are feasible. First, it is possible that based on the values of the numbers, connections with different strengths between the numbers are formed—numbers with closer values have stronger connections—and stronger connections create interference in a comparison task, thereby resulting in a distance effect. This explanation is similar to the ANS model in a sense that value-based semantic relations are responsible for the distance effect. As an alternative explanation, it is also possible that based on previous experiences, numbers are associated with the “small” and the “large” properties, e.g., large digits, such as 8 or 9, are more strongly associated with “large,” and small digits, such as 1 or 2, are more strongly associated with “small.” These associations could influence the comparison decision, and the number pairs with larger distance might be easier to process because the associations of the two numbers with the small-large properties differ to a larger extent. A similar explanation has been proposed earlier in a connectionist model, which model predicted several numerical effects successfully, and one key component of this model was that the distance effect relies on the connection between the number layer and the “larger” nodes, where relatively large numbers are associated with the “larger” node more strongly than relatively small numbers (Verguts et al., 2005).

Therefore, the explanations of the numerical distance effect suppose two different sources for the effect: According to the ANS model and to the value-based DSS explanation, the effect is rooted in the *values or the distance of the numbers*, whereas in the association-based DSS explanation and in the connectionist model, the effect is rooted in the *strength of the associations between the number and the small-large properties*. The two explanations are not exclusive; it is possible that both information sources contribute to the distance effect.

The two critical properties of the two explanations, i.e., the values or distance of the numbers and the association between the numbers and the small-large properties, strongly correlate in the number symbols used in everyday numerical tasks. Therefore, in those cases, one cannot specify their role in the distance effect. However, in a new artificial number notation, the two factors (the distance of the values and the association) could be manipulated independently. This is only possible if the distance effect is notation specific. Otherwise, the new symbols would get the association strengths of the already known numbers, instead of forming new association strengths between the new symbols and the small-large properties. It is possible that the numerical effects are notation specific, as has been already demonstrated in the case of the numerical size effect: In an artificial number notation comparison task, the size effect followed the frequency of the digits, which also means that the size effect is notation specific (Krajcsi et al., 2016).

## The aim of the study

**Figure 1:**
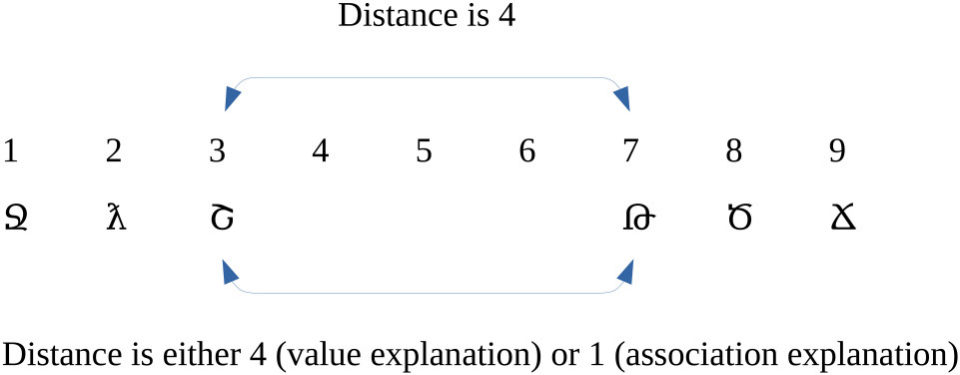
An example of the symbols and their meanings in the present study. Arrows show the predicted distance effect size based on the predictions of the two explanations.

The present study investigates whether in a new artificial number notation, where the values of the digits and the small-large associations do not necessarily correlate, the distance effect is influenced by the distance of the values or by the small-large associations, or both. One way to dissociate the two properties is to use a number sequence in which some of the values are omitted (Figure 1). If the distance effect is directed by the distance of the values, then the measured distance effect should be large around the gap (in this example, the effect should be measured as 4 units large), whereas if the distance effect is directed by the small-large associations, then the measured distance effect should be small around this gap, which is measured as an effect with a single unit distance, thereby supposing that the new digits were used in a comparison task with equal probability. If both mechanisms contribute to the distance effect, then the distance effect should be measured somewhere between the single unit and the many units (in this example 4 units) distance.

Why does the association explanation predict a distance effect of 1 distance around the gap? In a comparison task, the association between a digit and the small-large properties may depend on how many times the digit were judged as smaller or larger. If the new digits are used with equal probability in the comparisons (and if the distance effect is notation specific), then the probability of being smaller or larger than the other number can be specified easily (see Table 1). In our example (Figure 1), the number 1 is always smaller. Hence, the association frequency is 100% with the small property and 0% with the large property. The number 2 is smaller when compared with 3, 7, 8, and 9, and larger when compared with 1. Therefore, the association frequency is 80% small and 20% large. Continuing the example, the association frequency is directly proportional to the order of the symbols and not to their value. If the distance effect depends on the order, then the distance between 3 and 7 (i.e., the two digits around the gap) is the same as any other neighboring digits (see the specific values in Table 1).

**Table 1:** The chance of being smaller or larger in a comparison task when the symbols are presented with equal probability.

Example symbols	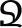	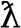	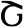	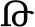	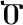	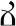
Meaning of the symbols	1	2	3	7	8	9
Chance of being smaller in a comparison	100%	80%	60%	40%	20%	0%
Chance of being larger in a comparison	0%	20%	40%	60%	80%	100%

**Figure 2:**
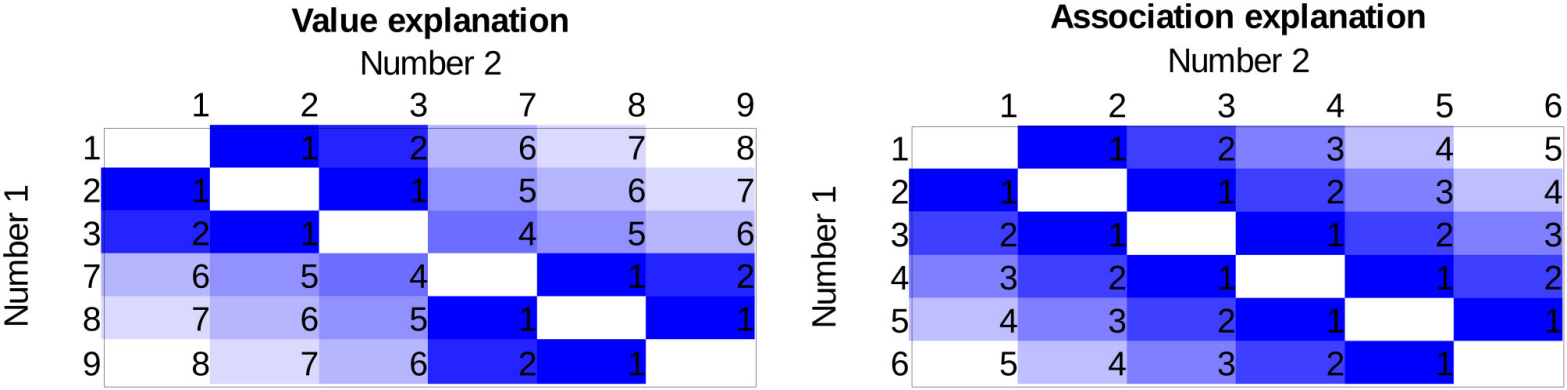
The expected distance effect pattern for the stimulus space used in the present study based on the value explanation **(left side)** and based on the association explanation **(right side)**. Specific values in the cells are the difference of the values (value model) or the difference of the order (association model) of the numbers to be compared on an arbitrary scale. Darker color indicates worse performance.

The two explanations predict different effect sizes for the distance effect not only for the two numbers next to the gap (e.g., for 3 vs. 7 on Figure 1 and Table 1) but also for any number pairs in which the two numbers are on the opposing side of the gap. The possible number pairs of the new symbols seen on Figure 1 and their hypothetical distance effect sizes according to the two explanations can be seen on Figure 2 Columns and rows denote the two numbers to be compared, and the cells show the distances of the value pairs (darker cells mean smaller distance). In the value explanation (left side), the predicted distance is the difference of the two numbers, whereas in the association explanation (right side), the predicted distance is computed based on the strength of the association with the small-large properties when the numbers are presented with equal probability, which is simply the order of those symbols in that series. The comparison performance should be proportional to the distance. Therefore, these figures show the performance pattern predictions according to the two explanations. The results will be displayed in a similar way as seen here because (a) displaying the full stimulus space is more informative than other indexes of distance effects, as any systematic deviation from the expected patterns could be observed, and (b) with the relatively large number of cells, any systematic pattern could be a convincing and critical information independent of the statistical hypotheses tests.

In the present test, it is critical that the new symbols should represent their intended values and not as a series that is independent of the intended number meanings; otherwise, the participants could consider the new symbols as numbers, e.g., from 1 to 6 because of their order in the new symbol series, which in turn could generate the performance predicted by the association explanation, even if the effect would be based on their values. One way to ensure that the new symbols are sufficiently associated to their intended values is to ensure that the priming distance effect works between the new and a well-known (for example, Indo-Arabic) notation. In numerical comparison tasks, the decision about the actual trial might be influenced by the stimulus of the previous trial, and the size of the influence is proportional to the numerical distance of the previous and actual stimuli, which is termed as the priming distance effect (PDE; Koechlin et al., 1999; Reynvoet and Brysbaert, 1999). The PDE is considered to be a sign of the relation between the symbols or the overlap of their representations (Opstal et al., 2008). Earlier experiments have shown that new artificial symbols can cause PDE in Indo-Arabic numbers (Krajcsi et al., 2016), suggesting that the new digits are not a series of symbols independent of their intended values, but they can be considered as a notation for the respective numbers. In the ANS framework, the PDE reflects the representational overlap between the numbers; thus, the PDE demonstrates that both notations appropriately activate the same representation—the ANS.

To summarize, the present study investigates whether the distance effect follows the distance of the values of the numbers (left of Figure 2) or the association of the small-large properties (right of Figure 2) or both, in the case of a newly learned notation (Figure 1), where some of the symbols are omitted. If both explanations are true, then we expect a pattern in-between the two figures, i.e., we should observe a break between 3 and 7 similar to the value explanation. However, the difference between the two sides of the gap should not be as large as in that explanation. All of these predictions only hold if the distance effect is notation specific; otherwise, the distance effect reflects the already well-known numbers, where the value and the association strongly correlates, and the pattern seen on the value model prediction can be expected. Consequently, only a pattern seen on the right in Figure 2 can decide about the models, because a pattern seen on the left can either mean a value-based distance effect or it can mean that the distance effect is notation independent.

## Methods

**Figure 3:**
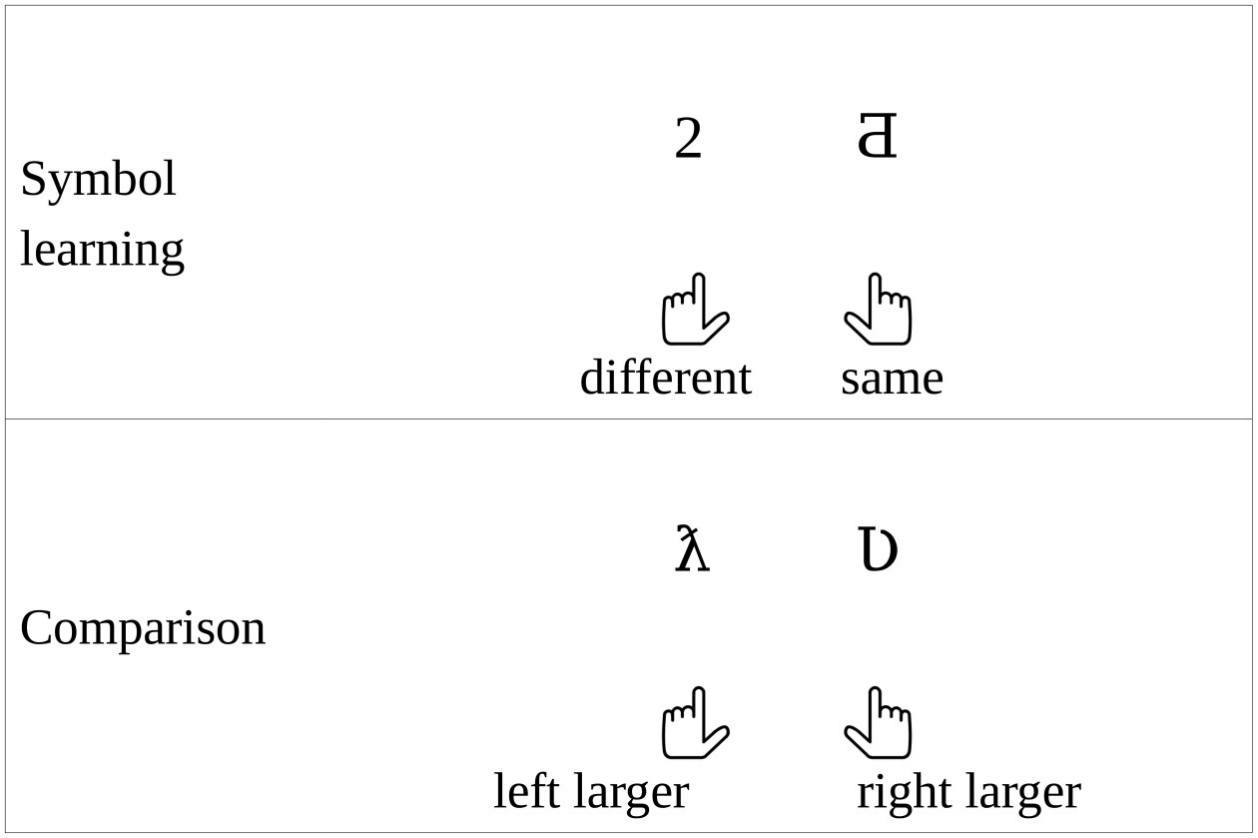
Tasks in the new symbol experiment.

In the present experiment, participants learned new symbols (Figure 3), with the meaning of the numbers between 1 and 3, and between 7 and 9 (Figure 1). Then a number comparison task was performed with the new symbols (Figure 3).

### Stimuli and procedure

The new symbols were chosen from writing systems that were mostly unknown to the participants (e.g., 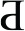, 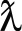, 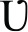, 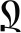). The characters had similar vertical and horizontal size, and similar visual complexity, and the height of the symbols were ~2 cm. (As mostly the apparent size does not influence the effects we investigate here, the visual angle was not controlled strictly.) Numbers were displayed in white on gray background. The symbols were randomly assigned to values for all participants, i.e., the same symbol could mean a different value for different participants.

The participants first learned new symbols for the numbers between 1 and 3, and between 7 and 9 (Figure 3). To ensure that the participants have learned them in the learning phase, symbols were practiced until a threshold hit rate was reached. In a trial, a new symbol and an Indo-Arabic digit were shown simultaneously, and the participant decided whether the two symbols denoted the same value by pressing the R or I key. The stimuli were visible until response. After the response, auditory feedback was given. In a block, all symbols were presented 10 times (60 trials in a block) in a randomized order. In half of the trials, the symbols denoted the same values. The symbol learning phase ended if the error rate in a completed block was smaller than 5% or the participant could not reach that level in five blocks.

In the following comparison task, the participants decided which number is larger in a simultaneously presented new symbol pair by pressing the R or I key (Figure 3). In a trial, two numbers were shown until response, and the participants chose the larger one. Numbers to be compared could be between 1 and 3, and between 7 and 9. After the response, auditory feedback was given. All possible number pairs including the applied numbers, excluding ties, were shown 15 times, thereby resulting in 450 trials.

Presentation of the stimuli and measurement of the responses were managed by the PsychoPy software (Peirce, 2007).

### Participants

Twenty-three university students participated in the experiment for partial course credit. After excluding 4 participants showing higher than 5% error rates (higher than the mean + the standard deviation of the error rates in the original sample) in the comparison task, the data of 19 participants was analyzed (16 females, mean age 22.2 years, standard deviation 4.6 years).

## Results

All participants successfully reached a lower than 5% error rate within 3 blocks in the symbol learning task. Therefore, no participants were excluded for not learning the symbols within 5 blocks.

**Figure 4:**
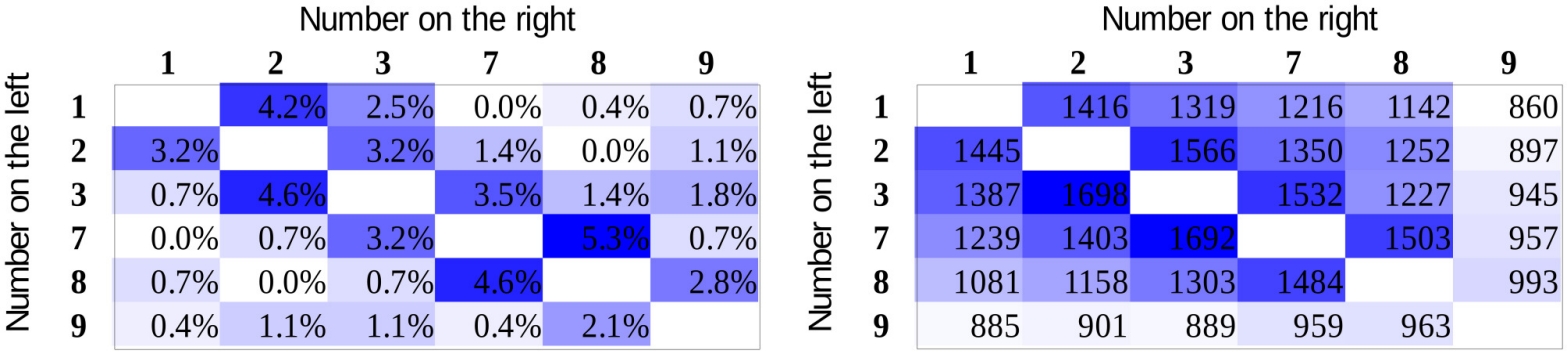
Error rates **(left)** and reaction times (in ms, **right**) in the whole stimulus space.

For all participants, the mean error rates and the mean reaction times for correct responses were calculated for all number pairs. Data of participants with higher than 5% mean error rate were excluded (higher than the mean + the standard deviation of the error rates in the original sample). The mean error rates and reaction times of the group are displayed in Figure 4 for the whole stimulus space. Visual inspection of the error rate pattern suggests that partly the value model can be observed, although the data are rather noisy, as reflected in some outlier cells. In the case of reaction time, it is more straightforward that the pattern is more in line with the association model (see the two expected pure patterns in Figure 2). In the reaction time data, one can also observe the end effect: number pairs including the largest number in the range (i.e., 9) are faster to process (Scholz and Potts, 1974; Leth-Steensen and Marley, 2000). (There are different possibilities concerning what causes the end effect. It is possible that participants learn that 9 is the largest number in the actual session; therefore, when 9 is displayed, no further consideration is required in a comparison task. Alternatively, according to the ANS model, it is possible that in the session, number 9 has neighboring number only on one side, and the overlap between the noisy signal distributions should be smaller, thereby leading to a faster response; Balakrishnan and Ashby, 1991).

To test the results statistically, we first fit the two predictions of the models (Figure 2) to the group average of the error rate and the reaction time data (Figure 4) with a simple linear regression, where one of the model predictions was the explanatory variable and one of the behavioral performance measurements was the dependent variable. Then the goodness of the fit measured as *R*^2^ was calculated (*R*^2^ columns given in Table 2), and the correlations of two models were compared with the method described by Steiger (1980) for every performance measurement (difference of the group fits column is given in Table 2). As an alternative method, we calculated the *R*^2^ values for every single participant for both the value and association models, and the *R*^2^ of these model fits, as ordinal variables, were compared pairwise with Wilcoxon signed-rank test (Better model for the participants column is given in Table 2).

**Table 2:** Goodness of fit of the models (measured as *R*^2^) and comparison of the correlations (Difference column) for the error rates, reaction times, and drift rates patterns based on the group average data, and hypothesis tests for choosing the better model based on the participants' data.

	**Linear model (Figure 2)**				**Logarithm model**			
	**Value model *R*^2^**	**Association model *R*^2^**	**Difference of the group fits**	**Better model for the participants**	**Value model *R*^2^**	**Association model *R*^2^**	**Difference of the group fits**	**Better model for the participants**
Error rate	0.709	0.708	*Z* = 0.008, *p* = 0.993	*T* = 73, *p* = 0.376	0.714	0.821	*Z* = −1.091, *p* = 0.275	*T* = 92, *p* = 0.904
Reaction time	0.543	0.790	*Z* = −2.294, *p* = 0.022	*T* = 44, *p* = 0.040	0.457	0.817	*Z* = −3.646, *p* < 0.001	*T* = 34, *p* = 0.014
Drift rate	0.526	0.861	*Z* = −3.647, *p* < 0.001	*T* = 39, *p* = 0.024	0.425	0.874	*Z* = −5.748, *p* < 0.001	*T* = 18, *p* = 0.002

To fit the distance effect appropriately, the end effect should also be considered, and its variance should be removed from the data. Inspection of the descriptive data on Figure 4 suggests that number pairs including the number 9 were involved in the end effect in the present study. One possibility to remove the end effect is to apply multiple linear regression, and beyond the distance effect regressor, an end effect regressor (e.g., 1 if the number pair includes 9, otherwise 0) also should be utilized. The problem with this solution is that the end effect not only shortened the response latency for number pairs including 9 but it also decreased the slope of the distance effect in those cells (see the less steep distance effect in the row and column with 9 than in other rows and columns). As the end effect is not added linearly to the distance effect, a multiple linear regression could not describe this nonlinear aspect of the end effect, which in turn would distort the distance effect results. As an alternative method, to remove the end effect, all cells with number pairs including 9 were removed from the analysis (i.e., the bottom row and the right column on Figure 4) and only the distance effect regressors were used. Therefore, for all linear fits (Table 2) in both the group average and the participants level, the number pairs including 9 were removed.

Regarding the possible difference between the goodness of fit of the two models, we note that the difference is limited by the fact that the two models correlate, e.g., the value model can be considered as a modified association model with an additional increase of the values in the top-right and bottom-left part of the stimulus space seen in Figure 2. Therefore, if one model is appropriate, then the other inappropriate model should show some non-zero *R*^2^ value too, although the *R*^2^ should be smaller than the *R*^2^ of the appropriate model.

Results for the goodness of fits (Table 2, linear model columns on the left) show that in the error rates, the two models are indistinguishable, and in the reaction time patterns, the association model seems to describe the data better in line with the visual inspection of the data.

**Figure 5:**
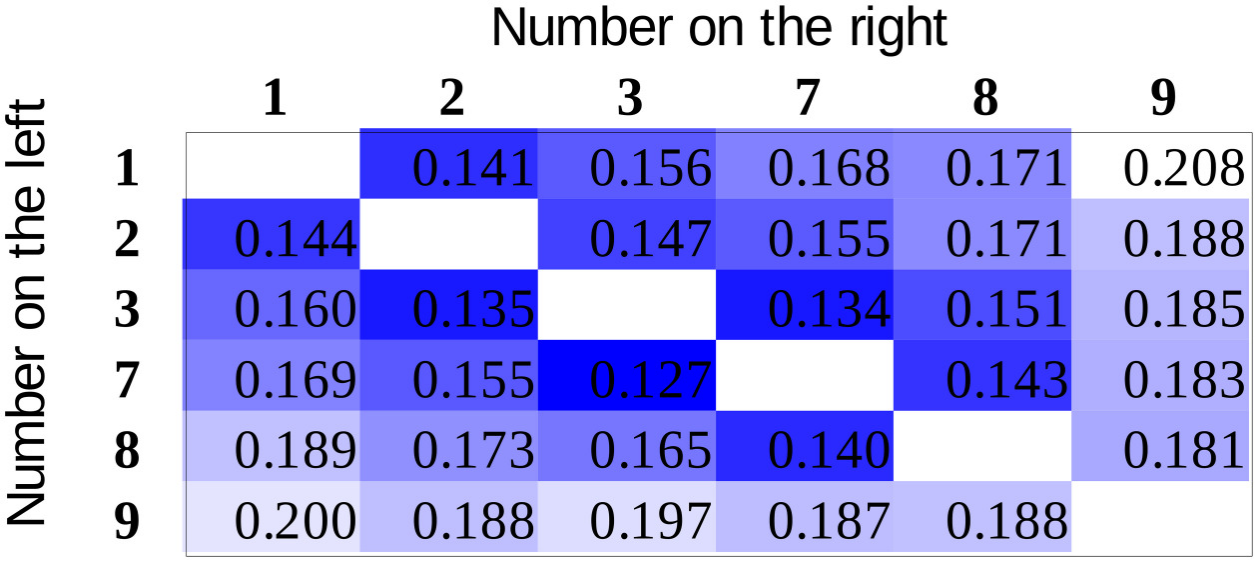
Drift rate values in the whole stimulus space.

Although error rate and reaction time data are highly informative, the recently becoming more popular diffusion model analysis could draw a more sensitive picture (Smith and Ratcliff, 2004; Ratcliff and McKoon, 2008). In the diffusion model, decision is based on a gradual accumulation of evidence offered by perceptual and other systems, and decision is made when appropriate amount of evidence is accumulated. Reaction time and error rates partly depend on the quality of the information (termed the drift rate) upon which the evidence is built. Drift rate is considered to be the most important parameter that influences the number comparison performance and the task difficulty (Dehaene, 2007). Importantly, observed reaction time and error rate parameters can be used to recover the drift rates (Ratcliff and Tuerlinckx, 2002; Wagenmakers et al., 2007). Drift rates can be more informative than the error rate or the reaction time because drift rates reveal the sensitivity of the background mechanisms more directly (Wagenmakers et al., 2007). To recover the drift rates for all number pairs, the EZ diffusion model was applied (Wagenmakers et al., 2007). The EZ model supposes that some of the parameters do not play a role in the response generation, and the model investigates and recovers only the drift rate, the decision threshold, and the non-decision time parameters. If one can suppose that only these three parameters play a role in the responses, then the EZ model can be utilized. Importantly, one essential advantage of this method is that unlike most other diffusion parameter recovery methods, EZ can be used when the number of trials per cells is relatively small. For edge correction, we used the half trial solution, i.e., for error rates of 0, 50, or 100%, the actual error rate was modified with the percent value of 0.5 trial, e.g., in a cell with 15 trials and 0% error rate, the corrected error rate was 0.5/15, which is 3.33% (see the exact details about edge correction in Wagenmakers et al., 2007). The scaling within-trials variability of drift rate was set to 0.1 in line with the tradition of the diffusion analysis literature. Drift rates for all number pairs and participants were calculated. The mean drift rates of the participants (Figure 5) show a similar pattern observed above for the former descriptive data. Fitting the two predictions of the models, the association model shows again a better fit (Table 2). In addition, (a) the largest difference between the goodness of fit of the two models can be observed for the drift rates (compared to the error rate and the reaction time data) and (b) the highest *R*^2^ value is found for the drift rates, thereby suggesting that the drift rate indeed captures the difficulty of the comparison tasks more sensitively than the error rates or the reaction times do.

The analysis above supposed that the distance effect (either coming from the value model or from the association model) is linear. However, a logarithmic or a similar function with decreasing change as the distance increases might be a better option to describe the data. First, one cannot suppose a linear distance effect, because after a sufficiently large distance, the reaction time should be unreasonably short or even negative, which would not make sense. Second, in a former artificial symbol comparison task, where the missing size effect did not influence the distance effect, the distance effect was better described with the logarithm function than with a linear function (unpublished results in Krajcsi et al., 2016). For these reasons, the analysis of goodness of fit was repeated with logarithmic distance effect models, in which the regressors were the natural logarithm of the values of the previously used linear models seen in Figure 2. The results (Table 2, logarithm model columns on the right) show that (a) for all three data types (error rate, reaction time, and drift rate), the association model fits better than it did with the linear regressor models and (b) the differences of the two models are larger than they were for the linear regressor models. Overall, the largest difference between the value and the association models can be seen in the logarithm model versions for the drift rates.

While our present main interest is the nature of the distance effect, it is worth to note that no size effect can be found in the data: The regressor formed as the sum of the two numbers to be compared (e.g., the regressor value for the 3 vs. 4 number pairs is 7) does not fit either the error rates (*R*^2^ = 0.001), or the reaction time (*R*^2^ = 0.01), or the drift rate (see below) data (*R*^2^ = 0.001). These data replicate the results of Krajcsi et al. (2016), thereby confirming that in new symbols with equal frequency of numbers in a comparison task, the size effect does not emerge and also confirm that the distance and size effects may dissociate. Relatedly, we note that the size effect could not influence the fit of the distance effect not only because the size effect could not be demonstrated in the present data but also because the size effect regressor (sum of the numbers to be compared) does not correlate with distance effect regressor (difference of the numbers to be compared) at all.

### Reliability of the results

**Figure 6:**
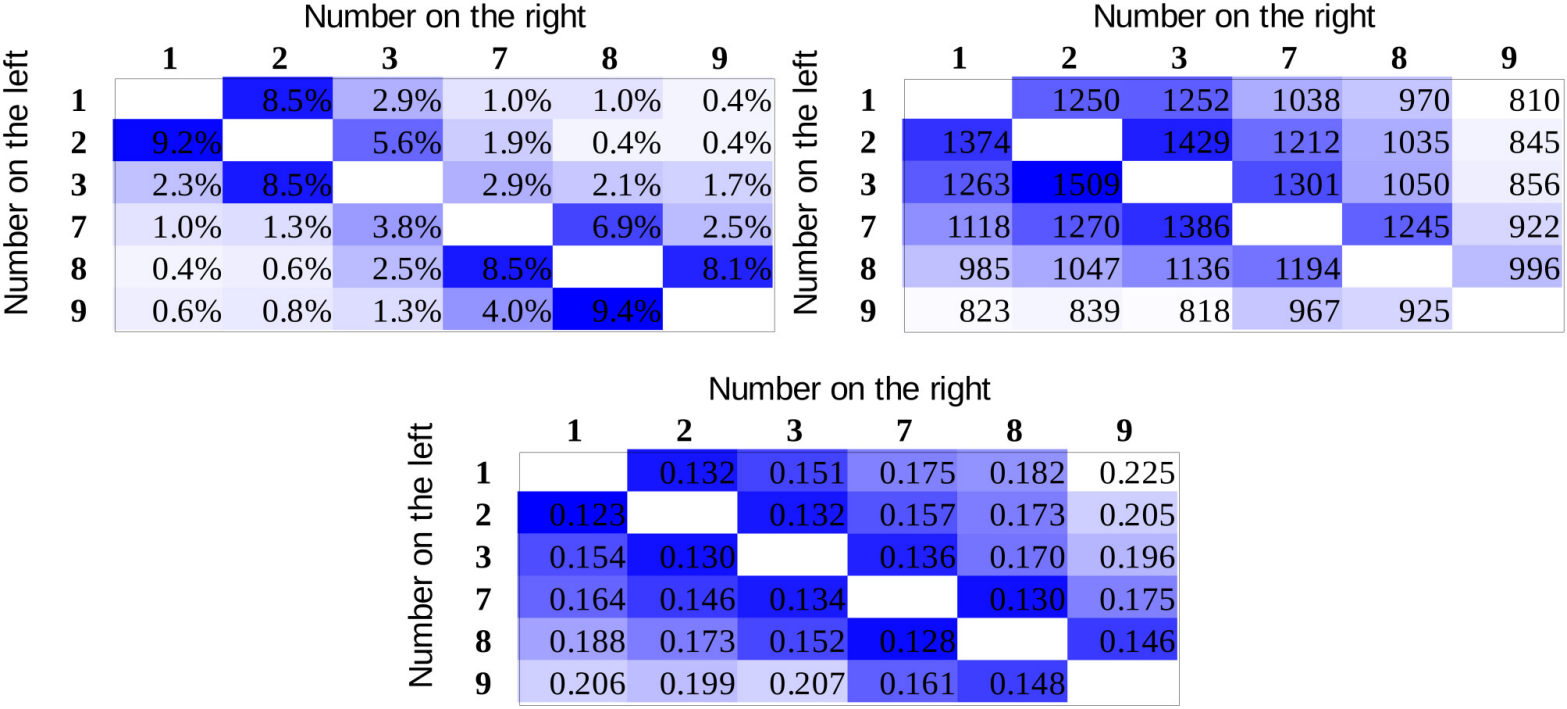
Error rates **(top left)**, reaction times (in ms, **top right**), and drift rates **(bottom)** in the whole stimulus space in the replication study.

To investigate the reliability of the present results, two additional experiments are summarized here: (a) the whole experiment was repeated with another sample and (b) the data of a follow-up study was analyzed where the same paradigm was used with Indo-Arabic numbers instead of new symbols to see if the distance effect can follow the associations of the numbers and small-large responses in an already well-established notation (Kojouharova and Krajcsi, Submitted). (a) In the replication study, 41 university students participated. Four of them were excluded, either because they did not reach the required maximum 5% error rate after 5 blocks of symbol learning or because they used wrong response keys. Five additional participants were excluded, because they had higher than 6.5% error rate (which was the mean + standard deviation error rate in that sample) in the comparison task. As a result, the data of 32 participants were analyzed (mean age was 21.0 years, 3 males). The error rate, reaction time, and drift rate means for the whole stimulus space can be seen in Figure 6, and the *R*^2^s of the models with the appropriate hypothesis tests are displayed in Table 3. While the reaction time and drift rate means replicate the results of the main study (although the difference was significant only with the comparison of the group fits, but not with the hypothesis test choosing the better fit for the participants), the error rates show the superiority of the value model. (b) In the Indo-Arabic comparison task, 23 university students participated. One participant was dyscalculic whose data were excluded from further analysis, and 2 further participants were excluded for having an error rate higher than 5%. Therefore, the data of 20 participants were analyzed (mean age was 20.15 years, 4 males). The goodness of fit of the logarithmic models and their contrast can be seen in Table 4. The Indo-Arabic study replicated the results of the main study, and also in the error rates, the association model fitted significantly better than the value model.

**Table 3:** Goodness of fit of the models (measured as *R*^2^) and comparison of the correlations (Difference column) for the error rates, reaction times, and drift rates patterns based on the group average data, and hypothesis tests for choosing the better model based on the participants' data in the replication study.

	**Linear model (Figure 2)**				**Logarithm model**			
	**Value model *R*^2^**	**Association model *R*^2^**	**Difference of the group fits**	**Better model for the participants**	**Value model *R*^2^**	**Association model *R*^2^**	**Difference of the group fits**	**Better model for the participants**
Error rate	0.791	0.629	*Z* = 2.041, *p* = 0.041	*T* = 130, *p* = 0.012	0.862	0.724	*Z* = 2.081, *p* = 0.037	*T* = 150, *p* = 0.033
Reaction time	0.610	0.719	*Z* = −1.258, *p* = 0.208	*T* = 233, *p* = 0.562	0.517	0.713	*Z* = −2.236, *p* = 0.025	*T* = 196, *p* = 0.204
Drift rate	0.768	0.914	*Z* = −2.727, *p* = 0.006	*T* = 232, *p* = 0.550	0.695	0.929	*Z* = −4.284, *p* < 0.001	*T* = 191, *p* = 0.172

**Table 4:** Goodness of fit of the models (measured as *R*^2^) and comparison of the correlations (Difference column) for the error rates, reaction times, and drift rates based on the group average data, and hypothesis tests for choosing the better model based on the participants' data in the Indo-Arabic study (Kojouharova and Krajcsi, Submitted).

	**Logarithm model**			
	**Value model *R*^2^**	**Association model *R*^2^**	**Difference of the group fits**	**Better model for the participants**
Error rate	0.634	0.825	*Z* = −2.766, *p* = 0.006	*T* = 17, *p* = 0.001
Reaction time	0.749	0.917	*Z* = −3.737, *p* < 0.001	*T* = 14, *p* < 0.001
Drift rate	0.681	0.864	*Z* = −3.080, *p* = 0.002	*T* = 31, *p* = 0.006

Looking strictly at the significance of the results, the replication shows a somewhat different result pattern as the first measurement, because in error rate, the significant differences support the value model instead of the association model, and in reaction time and drift rate, not all hypothesis tests are significant. Clearly, some non-significant effects might reflect not only due to the lack of an effect but also due to the lack of statistical power, and significant effects can also be type-I errors (there is especially a chance for this, when replication studies find opposing significant effects). To evaluate the accumulated data, a mini meta-analysis was run on the three set of data (Maner, 2014). Binary random-effects with the DerSimonian-Laird method (Viechtbauer, 2010; Wallace et al., 2012) was performed on the logarithm model fit data measuring the ratio of participants where the association model was better than the linear model. While the error rate does not show a clear preference for any models (45.9% mean preference for the association model with 95% CI of [17.5, 74.2%]), reaction time and drift rate clearly prefer the association model (76.6% with CI of [65.0, 88.2%] for reaction time and 72.9% with CI [58.2, 87.7%] for drift rate). Taken together, while the reaction time and drift rate show the superiority of the association model, the results of the error rates are ambiguous. It is important to highlight that from the viewpoint of the present question, reaction time and especially drift rates are more relevant. First, reaction time data are usually considered to be more reliable and sensitive than error rate, because error rate and reaction time data measure two strongly correlating constructs. Error rate measures it in a dichotomous scale, whereas reaction time is a continuous scale. Therefore, the latter have more information about the trial performance. Second, drift rate measures the difficulty of the task more sensitively than error rates or reaction times in themselves (Wagenmakers et al., 2007; this is also confirmed by the usually higher *R*^2^ values for drift rates than for reaction times or error rates). Therefore, we consider that reaction times and drift rates reliably reflect the superiority of the association model over the value model. At the same time, it might be a question of future research whether heterogeneous error rates are the result of random noise or whether there are aspects of performance that partly reflects the functioning of the value model.

To summarize the results, it was found that (a) the association model described the distance effect better than the value model; it measured with reaction time and drift rate, while error rate displayed an inconsistent pattern, (b) drift rate draws more straightforward picture than the reaction time or the error rate data, (c) logarithmic type distance effect describes the data more precisely than the linear distance effect, and finally, (d) size effect is absent in the present paradigm with uniform number frequency distribution.

## Discussion

The present work investigated whether the numerical distance effect is rooted in the values of the numbers to be compared or in the association between the numbers and the small-large properties. In a new artificial number notation with omitted numbers, the distance effect measured with reaction time and drift rate did not follow the values of the numbers, as it would have been suggested in the mainstream ANS model (Moyer and Landauer, 1967; Dehaene, 2007) or in the value-based explanation of the DSS model. Instead, the effect reflected the association between the numbers and the small-large categories, as proposed by the association-based explanation of the DSS model or by the delta-rule connectionist model of numerical effects (Verguts et al., 2005). Measured with error rate, the results were not conclusive, so it is the question of additional studies whether the inconsistency in the error rate data is simply noise or there are additional aspects of the distance effect that should be investigated with more sensitive methods.

Together with the present results, several findings converge to the conclusion that the symbolic number comparison task cannot be explained by the ANS. First, unlike the prediction of that model suggesting that distance and size effects are two ways to measure the single ratio effect, symbolic distance and size effects are independent (Krajcsi, 2017), and the distance effect can be present even when no size effect can be observed (shown in the present results and in Krajcsi et al., 2016). Second, the size effect follows the frequency of the numbers as demonstrated in Krajcsi et al. (2016) and also in the present results, where the uniform frequency of the digits induced no size effect (i.e., the slope of the size effect is zero). Third, the present data demonstrated that the distance effect is not directed by the values of the digits as predicted by the ANS model, but they are influenced by the frequency of the association with the small and large categories (see also the extension of the present findings for Indo-Arabic numbers in Kojouharova and Krajcsi, Submitted).

The present and some previous results also characterize the symbolic numerical comparison task; an alternative model should take the following into consideration: (a) symbolic distance and size effects are independent (Krajcsi et al., 2016; Krajcsi, 2017), (b) the effects are notation independent (the present results and Krajcsi et al., 2016), (c) the size effect depends on the frequency of the numbers (the present results and Krajcsi et al., 2016), (d) the distance effect depends on the association between the numbers and the small-large categories (present results), and (e) the distance effect can be described with a logarithm of the difference of the values (present results).

It is again highlighted that these results are not the consequence of the possibility that the new symbols are not related to their intended values and that the independent series of symbols would create a performance pattern similar to the association model prediction, because it was already shown that the new symbols prime the Indo-Arabic numbers, thereby revealing that the new symbols denote their intended values (Krajcsi et al., 2016). The present findings were also replicated with Indo-Arabic numbers (Kojouharova and Krajcsi, Submitted).

From a methodological point of view, it is worth to note that in the present comparison task, the drift rate seemed to be the most sensitive index to describe performance, which strengthens the role of the diffusion model analysis, among others in cases when sensitivity and statistical power are essential.

To summarize, the results revealed that in an artificial number notation where some omitted numbers might create a gap, the distance effect followed the association with the small-large properties and not the values of the numbers. This result contradicts the Analog Number System model and the value-based DSS explanation, which suggests that the distance effect is directed by the values or the ratio of the numbers. On the other hand, the result is in line with the alternative association-based DSS explanation and the delta-rule connectionist model, in which the distance effect is directed by the association between the number nodes and the small-large nodes.

## Ethics statement

All studies reported here were carried out in accordance with the recommendations of the Department of Cognitive Psychology ethics committee with written informed consent from all subjects. All subjects gave written informed consent in accordance with the Declaration of Helsinki.

## Author contributions

All authors listed have made a substantial, direct and intellectual contribution to the work, and approved it for publication. Both authors contributed equally to this work.

### Conflict of interest statement

The authors declare that the research was conducted in the absence of any commercial or financial relationships that could be construed as a potential conflict of interest.
